# A framework for distributed health professions training: using participatory action research to build consensus

**DOI:** 10.1186/s12909-020-02046-z

**Published:** 2020-05-14

**Authors:** Susan C. Van Schalkwyk, Ian D. Couper, Julia Blitz, Marietjie R. De Villiers

**Affiliations:** 1 Centre for Health Professions Education, Faculty of Medicine and Health Sciences, Stellenbosch University, Stellenbosch, South Africa; 2 Ukwanda Centre for Rural Health, Department of Global Health, Faculty of Medicine and Health Sciences, Stellenbosch University, Stellenbosch, South Africa; 3 Division of Family Medicine and Primary Care, Faculty of Medicine and Health Sciences, Stellenbosch University, Stellenbosch, South Africa

**Keywords:** Distributed training, Health professions training, Policy implementation, Participatory action research, Complexity theory

## Abstract

**Background:**

There is a global trend towards providing training for health professions students outside of tertiary academic complexes. In many countries, this shift places pressure on available sites and the resources at their disposal, specifically within the public health sector. Introducing an educational remit into a complex health system is challenging, requiring commitment from a range of stakeholders, including national authorities. To facilitate the effective implementation of distributed training, we developed a guiding framework through an extensive, national consultative process with a view to informing both practice and policy.

**Methods:**

We adopted a participatory action research approach over a four year period across three phases, which included seven local, provincial and national consultative workshops, reflective work sessions by the research team, and expert reviews. Approximately 240 people participated in these activities. Engagement with the national department of health and health professions council further informed the development of the Framework.

**Results:**

Each successive ‘feedback loop’ contributed to the development of the Framework which comprised a set of guiding principles, as well as the components essential to the effective implementation of distributed training. Analysis further pointed to the centrality of relationships, while emphasising the importance of involving all sectors relevant to the training of health professionals. A tool to facilitate the implementation of the Framework was also developed, incorporating a set of ‘Simple Rules for Effective distributed health professions training’. A national consensus statement was adopted.

**Conclusions:**

In this project, we drew on the thinking and practices of key stakeholders to enable a synthesis between their embodied and inscribed knowledge, and the prevailing literature, this with a view to further enaction as the knowledge generators become knowledge users. The Framework and its subsequent implementation has not only assisted us to apply the evidence to our educational practice, but also to begin to influence policy at a national level.

## Background

As the body of knowledge around health professions education (HPE) research continues to grow, questions are being asked about its potential to influence policy and enhance practice. In as early as 2000, Van der Vleuten and colleagues [1] issued a plea that the evidence being generated in the field, be applied in our educational practice. Additionally, the World Health Organization has called for the scaling up of health professions education, while acknowledging there are low levels of evidence for the recommended ways to improve the quantity, quality and relevance of that education [2]. We support these calls, and further argue that such application should, where appropriate, not only have relevance for our day to day teaching, but also influence policy and practice at national level given that in many countries, the training of health professionals is bound by educational and health legislative frameworks. As new evidence about teaching, learning and assessment emerges, HPE researchers should consider how this evidence could be generated in such a way for it to be regarded by national authorities and decision-making bodies as both credible and valid, thereby creating the potential for such evidence to influence practice. Furthermore, in thinking through the critical and creative process of linking knowledge and practice, we need to move beyond the notion of knowledge translation [3] to greater recognition and embodiment of knowledge co-production [4]. At the same time, we acknowledge that mobilising knowledge for both policy and practice is a complex and context-dependent process [5], leading to difficulties with achieving partnership in evidence generation [6].

In 2015, we embarked on a project to develop a framework for effective distributed health professions training (DHPT) in South Africa (SA). The term ‘decentralised’ was used at the outset of the project, but was replaced over time with the term ‘distributed’. As the work evolved, we came to see ‘decentralised’ as having oppositional undertones, whereas ‘distributed’ was felt to reflect a more open and non-hierarchical approach. Distributed training we described as ‘training activities for undergraduate [HPE] students that take place away from tertiary academic complexes – for example: health care centres, primary care clinics and district and rural hospitals’. [7] Such training provides opportunity for students to be directly exposed to local health contexts, the social determinants of health, the continuum of comprehensive care and the role of context in health and illness, with the potential to address the maldistribution of human resources for health [8]. Our intention was to generate a body of evidence that would have value for institutions that send students for clinical placements outside of the central academic hospital and inform the development of the envisaged framework. From the outset, however, we were also aware that distributed training could not occur without support and commitment from national authorities, and that for the framework to have applicability beyond a local or institutional context, a national consensus would be needed which included these authorities in its development.

In this article, we share our Framework for DHPT. We describe why such a framework is necessary, and detail the approach that was followed in developing it, including how we intentionally sought to ensure national relevance. Finally, we offer critical reflection on the process of knowledge generation with a view to influencing policy that might ultimately influence practice at the national level. While this work was undertaken in SA, and notwithstanding our in-country context, we believe that both the Framework and our approach to knowledge generation across a national community will resonate with others in Africa and around the globe.

### Why DHPT and the need for a framework?

The development of a framework for DHPT was prompted both by international and national developments. The oft-cited calls for transformation of health professions education [2, 9] have included decentralisation/distribution of this training as a key element. Linked to this is an understanding of the importance of social accountability that requires graduates to be equipped in responding to the needs of the communities they serve [10, 11]. The vital role of context in driving these changes is increasingly recognised [12]. HPE literature on distributed training is rapidly increasing describing many successful initiatives in a number of countries, including sub-Saharan and South Africa [12–15]. Motivating factors in the development of such DHPT include workforce effects in terms of the scaling up of training referred to above; educational advantages arising from distributing students; and the benefits afforded by having students training at these health facilities [16].

In SA, an inadequate number of health professions schools that are largely hospital-based and structured around specialist silos, produce too few graduates for the country’s needs [17, 18]. In a recent landmark report on reconceptualising health professions education, the Academy of Science of SA recommends inter alia that ‘training of health professions students should be orientated towards addressing inequity and meeting the needs of the most underserved, through supporting a primary care focus and increasing the supply of health care workers to rural areas’. [19] Moreover, national government plans to address universal health coverage through a national health insurance (NHI) scheme that will demand both greater numbers of health professionals to be trained and graduates who are better able to work in primary care, district health services and communities, with a focus on the quadruple burden of disease faced by the population [20].

A response to the imperatives listed above has been an increasing uptake of distributed training for undergraduate students. However, these growing numbers place pressure on the sites available for such clinical training, specifically within the public health sector [8], with institutions competing for placements while individually, or sometimes regionally, seeking to negotiate with local and regional health authorities in this regard. As initiatives by the different training institutions facilitated progress in extending distributed training activities, we believed that the development of a framework to support the implementation of DHPT at a national level that could foster coherence across the system would have value. We therefore embarked on a national consultative process that drew on existing evidence, and the knowledge and expertise of a wide range of role-players, to establish a tool that could inform practice at institutional, local, regional and national level. Conceptually our work was based on an assumption that those within the sector – the community of healthcare professionals, including educators, who work within or in support of the distributed sites – were well-positioned to develop the framework in a collaborative and participatory manner thus paving the way for the methodology that is described below. We drew on complexity theory as a basis for our work, acknowledging the complex nature of the environments within which DHPT occurs. Using this lens, we understood complexity to be an innate characteristic of a system, whose properties emerge from the relationships of the components in that system [21]. Our over-arching research question was therefore designed specifically to facilitate a wide range of inputs and contributions: what should a framework for the implementation of DHPT look like?

## Method

This work was undertaken over a period of four years (2015–2018), comprised multiple data collection and generation activities, and included a large number of participants from across the country. Our research team included an educationalist, three family physicians, and an obstetrician/gynaecologist. We have all been active in health professions education, including community-based education and rural health, for many years. It was inevitable therefore that many, if not all, of those who participated in this study were known to at least one of us. This facilitated recruitment and also, we believe, enhanced the quality of engagement during the different data generation events, as there were pre-existing relationships and mutual understandings of context. However, as ‘insider researchers’ we were aware of the need to be reflexive, cognisant of our assumptions and expectations, treating our participants ethically in seeking to accurately capture their responses in our writing [22].

We adopted a participatory action research (PAR) approach based on iterative cycles (phases) of reflection, data collection and action [23]. PAR has social transformation as its philosophical foundation, and has been defined as a ‘democratic process concerned with developing practical knowing in the pursuit of worthwhile human purposes’ [24] Given our stated intention to move towards national consensus, to influence practice and policy, and to ultimately benefit health care in the country, this approach was well-suited to our needs. Healthcare has been described as a ‘complex adaptive system’ and we realised that PAR would allow for a process of ‘feedback loops’ that would maintain the momentum for change [25]. We further acknowledged the potential of PAR in the context of multi-disciplinary research that seeks to link theory to practice [26]. Kemmis et al. [27] have argued that PAR is premised on enabling practitioners who share a particular discourse to engage in meaningful debate around their own practice. It allows for transforming ‘the conduct and consequences of their practice … from within’. We specifically adopted the approach described by Reason and Bradbury [24] which emphasizes collective inquiry grounded in the experience of the participants, forming communities of inquiry that address questions and issues that are significant for those who participate. We sought to integrate the three basic aspects of participation, action, and research using several cycles of knowledge generation [28].

The developmental process, from conceptualisation to implementation, comprised three phases that included seven local, provincial and national, consultative workshops focusing on facilitating stakeholder input and dialogue reflective work sessions by the research team, as well as expert review. Approximately 240 people participated in the activities and initiatives that informed the development of the Framework and implementation tool between 2015 and 2018. Table 1 shows who the workshop participants were, how many attended per workshop, and the purpose and outcomes of each workshop. The dates in Table 1 provide a sense of the progression of the work over time. Stakeholder engagement with the National Department of Health (NDoH) and the Health Professions Council of South Africa (HPCSA) took place in February and March 2017. In keeping with the PAR process, analysis was undertaken throughout the course of the project. Ethics approval was received from the Stellenbosch University Faculty of Medicine and Health Science Research Ethics Committee # N16/03/034, as well as the funder.

**Table 1:** The seven consultative workshops

#	Where	When	Who	How many	Purpose	Outcomes
Phase 1 – Establishing the foundation						
1	Cape Town, Western Cape,	October 2015	Medical schools; and Department of Health representatives	33	Initiate process to develop a framework for DHPT in undergraduate medical training	Current practice in DHPT in SA
						Key factors for enabling DHPT
						Priorities, gaps and challenges in DHPT
						Visual models [7]
2	Port Elizabeth, Eastern Cape	June 2016	Range of health professions; Deans’ representatives	28	Provide opportunity for multi-professional engagement in developing a DHPT framework	Definition of DHPT
						Vision statement Components of vision
Phase 2 – Developing the Framework and the Implementation Tool						
3	Cape Town, Western Cape	June 2017	Faculty members	25	Stakeholder input	Verification of enabling factors
4	Potchefstroom, North West	July 2017	Range of health professions)	41	Develop strategies for effective DHPT	Challenges, barriers and bridges in DHPT
						Validation of enabling factors
						Consensus statement on DHPT
						Formation of Special Interest Group Framework for DHPT
Phase 3 – Implementing and refining the Framework						
5	Durban, KwaZulu-Natal	June 2018	Range of health professions	28	Enable participants to implement the DHPT framework in their local context	Implementation tool (Annexure A) piloted
						Workshop format to use the implementation tool trialled
6	Mthatha, Eastern Cape	September 2018	Faculty members, clinical supervisors, students	35	Evaluate WSU DHPT programme using the implementation tool	Implementation tool refined
7	Durban, KwaZulu-Natal	September 2018	Faculty members	14	Use the implementation tool	Framework and tool applied a in another context.

## Results

### Phase 1 – establishing the foundation

As a foundation for the PAR activities, the research team conducted a scoping review to identify approaches to distributed training as found in current literature. This review provided an over-arching perspective on the topic, offering a first level of evidence to inform the development of the Framework. It identified student learning, the training environment, the role of community and leadership and governance as essential components for DHTP [14]. Workshop 1 formally initiated the process of participatory work. During this two-day event, participants representing all nine medical schools in SA, described current practice across their different institutions, and identified priorities, gaps and challenges for implementing DHPT. The group further proposed a preliminary set of key factors for enabling DHPT, clustered around the essential components that had been identified from the scoping review [7]. A year later, Workshop 2 engaged with these sets of ideas again, looking to concretise a definition and develop a vision statement for DHPT (Table 2), which was premised on a set of guiding principles (Table 3). This second workshop expanded the reach of the project by extending an open invitation to all interested health professionals who were in some way or another involved in the clinical training of HPE students.

**Table 2:** A Vision for effective DHPT

Effective DHPT facilitates learning that is transformative, reflective, socially accountable, community-engaged, self-directed, inter-professional, collaborative and peer-to-peer. The curriculum for distributed training is relevant, primary health care oriented, holistic, fit for purpose, and delivered in an integrated, continuous and longitudinal manner. Sufficient resources are made available for distributed training. Teachers and supervisors are motivated and suitably equipped for their task. Students embrace distributed learning.

**Table 3:** Guiding principles

**A shared vision**: all stakeholders across all levels recognise the need to work towards a shared vision for distributed training as a catalyst for good quality, relevant health professions training addressing the health care and human resources needs of the country.	
**Social accountability**: orientating the training of students towards the health needs of the community in order to foster the development of socially accountable health care workers who are motivated to work in underserved areas once qualified.	
**Continuity:** immersed, longitudinal, distributed training in and with communities, fostering continuity of learning and relationships with health services, managers, health care teams, trainers, staff, training institution(s), students, patients/clients and the community.	
**Responsive adaptability**: continuous renewal and adaptation of curricula, training methods and approaches, ensuring they are flexible but achieve educational equivalence in different settings so as to be responsive to community and health system needs.	
**Integration:** fostering an integrated approach to learning, a curriculum that merges clinical and public health approaches and promotes inter-professional learning and collaborative practice across disciplines and various levels of care.	

The research team subsequently held a two-day reflective work session during which the findings of the scoping review, the key factors and visual artefacts (Workshop 1) and the vision for DHPT (Workshop 2), were revisited and synthesised through a process of shared critical reflection and sense making. Thematic analysis of the artefacts developed during or as a result of the workshops (group notes, reports, drawings, etc.), was also undertaken, using a process of individual review during which team members separately developed a set of codes, followed by collaboratively categorising these into over-arching themes. (For examples of artefacts see De Villiers et al. 2017) [7]. This interpretive synthesis generated an expanded set of enabling factors (arising from the key factors identified in the scoping review) for effective DHPT that could inform the operationalisation of the essential components [7]. These were subjected to an expert review process (with 21 representatives from academic institutions and 12 from the health services) conducted electronically in May 2017, and were revised accordingly.

### Phase 2: developing the framework and the implementation tool

Workshops 3 and 4 were held during this phase where participants reflectively engaged with the revised set of essential components and the enabling factors. The Adaptive Action approach of Eoyang and Holladay [29], an iterative planning process arising from complexity theory, was introduced to facilitate further refinement resulting in the final set of 41 enabling factors to facilitate the implementation of effective DHTP (Table 4).

**Table 4:** Essential components and their enabling factors (this is an abbreviated version, please see Additional file 1 for the unabridged version)

Leadership and governance influences effective DHPT, through the decision-making processes and roles and responsibilities of stakeholders.	
1. Stakeholders engage in partnerships.	
2. Roles and responsibilities of stakeholders are clear..	
3. Management is committed to collaboration..	
4. Stakeholders’ senior management demonstrate visionary leadership.	
5. Champions take responsibility for distributed training.	
6. Funding is made available.	
7. Communication channels exist among stakeholders.	
8. Monitoring, evaluation, and research are encouraged by leadership.	
9. The training institution:	
•implements institutional policies supporting distributed training.	
•capacitates primary supervisors and other site staff.	
•maintains relationships with the site.	
•selects students most likely to practice in distributed areas.	
•is familiar with the each site’s strengths and challenges.	
The curriculum provides the scaffolding that informs the learning outcomes, content, mode of delivery, and assessment of students, and evaluation of the curriculum itself.	
10. Management prioritises distributed training.	
11. Learning outcomes across training institutions are consistent.	
12. Learning outcomes for distributed training include a focus on:	
•Social determinants of health.	
•Common, undifferentiated problems in primary health care.	
•An integrated spectrum of health and illness.	
•Cultural awareness.	
13. The curriculum for distributed training uses:	
•Various teaching and learning approaches.	
•A patient-centered approach to care.	
•Opportunities for developing a range of competencies.	
•Adaptability to the realities of the individual site.	
•On-site, integrated and continuous student assessment.	
14. Rotations should be of sufficient length to allow for student immersion.	
15. Students provide and receive regular feedback.	
16. Monitoring, review, and modification of the curriculum is performed.	
The community is defined as the population that utilises the local health facility where students are trained, and is the reference point for the curriculum.	
17. Community stakeholders are engaged.	
18. Partnerships are maintained with community stakeholders.	
19. The community shares the vision for training.	
20. Students and staff are aware of community needs.	
21. Learning opportunities are available in the community.	
22. Students learn through being immersed in the community.	
23. Stakeholders engage in celebration of accomplishments.	
The training environment includes (a) people who work at the distributed training site, and in the community, contributing to the training of the students; and (b) the training site as the context and physical environment within which the distributed training takes place.	
(a) People	
24. A dedicated person coordinates the training at the site.	
25. Staff from various professions work with students to facilitate their learning.	
26. Site staff receive guidelines to support students’ learning.	
27. Site staff receive recognition from the training institution.	
28. Site staff provide feedback about student performance.	
29. Subject specialists support distributed training through regular outreach visits.	
30. At least one health professional acts as primary supervisor for students.	
31. The primary supervisor:	
•develops, implements, and evaluates the training at the site.	
•is involved in assessment of students.	
•receives the necessary support and training technologies.	
•develops capacity in teaching and learning.,	
(b) Place	
32. The training site is selected collaboratively by stakeholders.,	
33. Site selection is based on factors that facilitate relevant learning opportunities.	
34. Medical equipment, appropriate to the level of care, is available.	
35. Sufficient space for training activities is made available.	
36. Materials to enhance learning are made available on-site.	
37. Accommodation and transport for students are made available.	
The students are learners enrolled for any programme in health professions at a training institution.	
38. Students:	
•receive orientation before they begin a rotation.	
•have academic and social support available.	
•provide feedback after they complete a rotation.	
•have adequate arrangements for safety and security.	
39. Student-staff ratios are mutually agreed upon.	
40. At least two students are assigned to a site.	
41. Reasonable logistical arrangements are made by the training institution.	

**Fig. 1:**
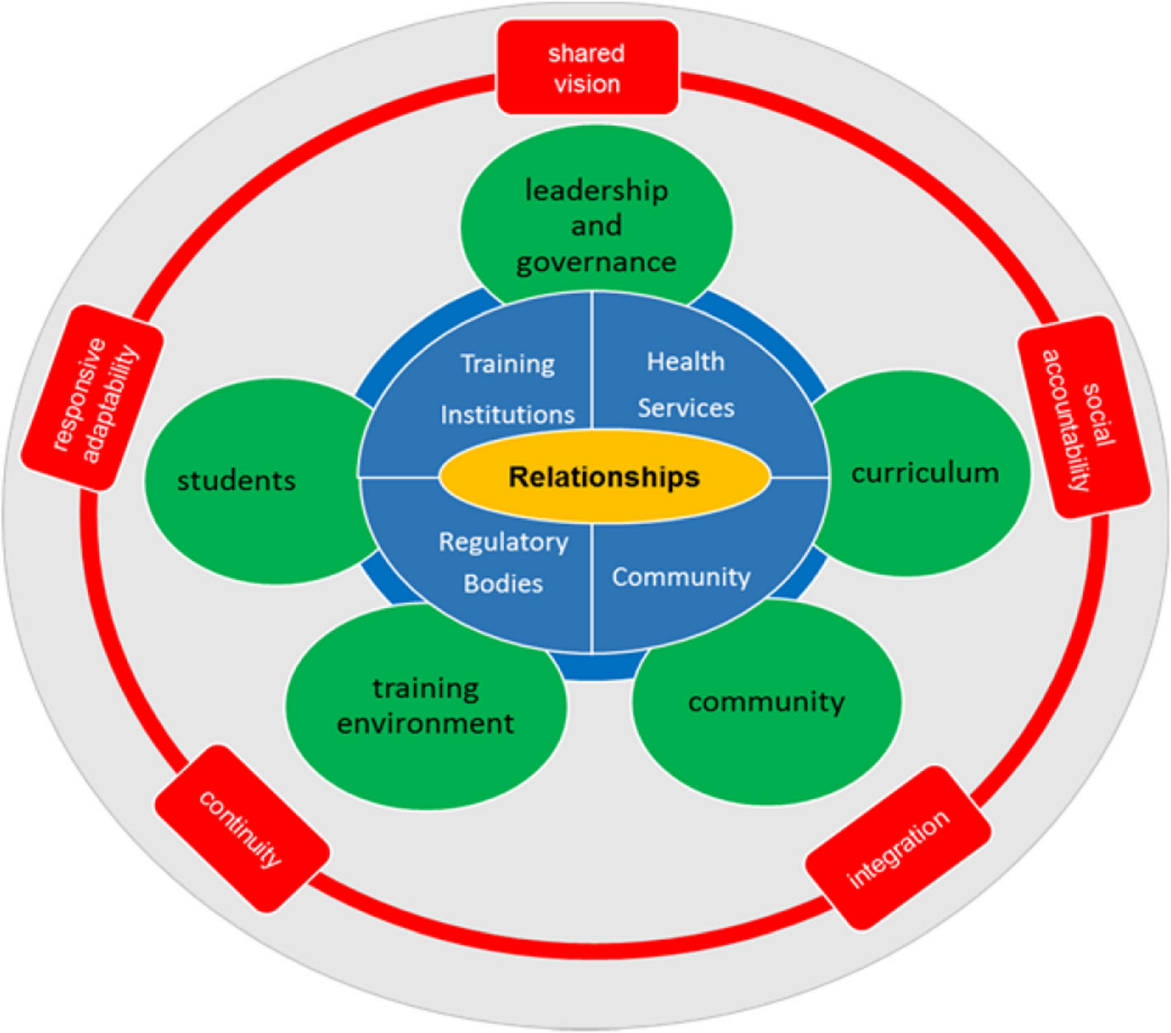
Framework for effective DHPT. The framework comprises guiding principles in red and the essential components in green. Relationships are central and placed at the heart of the framework in yellow with stakeholder engagement in blue

A further outcome from Workshop 4 was the approval of a national consensus statement for DHPT, developed by the research team, which was then adopted by the South African Association of Health Educationalists at their annual conference in June 2017 [8] (http://saahe.org.za/2017/07/consensus-statement-on-decentralised-training-in-the-health-professions/) and led to the establishment of a Special Interest Group for DHPT within the organisation. The consensus statement has subsequently been endorsed by at least 11 professional bodies and training institutions representing a significant proportion of the key role-players in HPE in South Africa. It was also at Workshop 4 that the Framework for effective DHPT (Fig. 1) was conceptualised comprising the guiding principles and the essential components. Analysis had further pointed to the centrality of relationships and hence its placement at the heart of the framework while also emphasising the importance of wide stakeholder engagement, involving all sectors relevant to training health professionals in the country, to ensure the sustainability of programmes long term.

Throughout this phase, the research team continued to adopt a critically reflective process during regular project meetings, thus ensuring iterative cycles of data collection, reflection and action, while drawing on their own lived experiences and that of their participants [26]. A key realisation during this process was the need to facilitate the implementation of the Framework. This led to a final outcome from Phase 2 namely the Implementation Tool, which again drew on the principles of Adaptive Action and saw the reorganisation of the enabling factors into a set of ‘Simple Rules for Effective DHPT’ (Annexure A). This Tool was intended to guide the implementation of new programmes, assess and improve existing programmes, and engage stakeholders in sustainable design, implementation, and evaluation of distributed training initiatives.

### Phase 3: implementing and refining the framework

During this final phase, the Implementation Tool was piloted during a series of workshops (5–7), while evolving drafts of the Framework and the Tool were presented at a number of national and international conferences and other events to key stakeholders (NDoH, HPCSA). This allowed for further refinement based on contextual input, thereby sustaining engagement and interest, and the identification of those elements of the tool which would be most useful in practice [30]. Further engagement with local communities is envisaged.

**Fig. 2:**
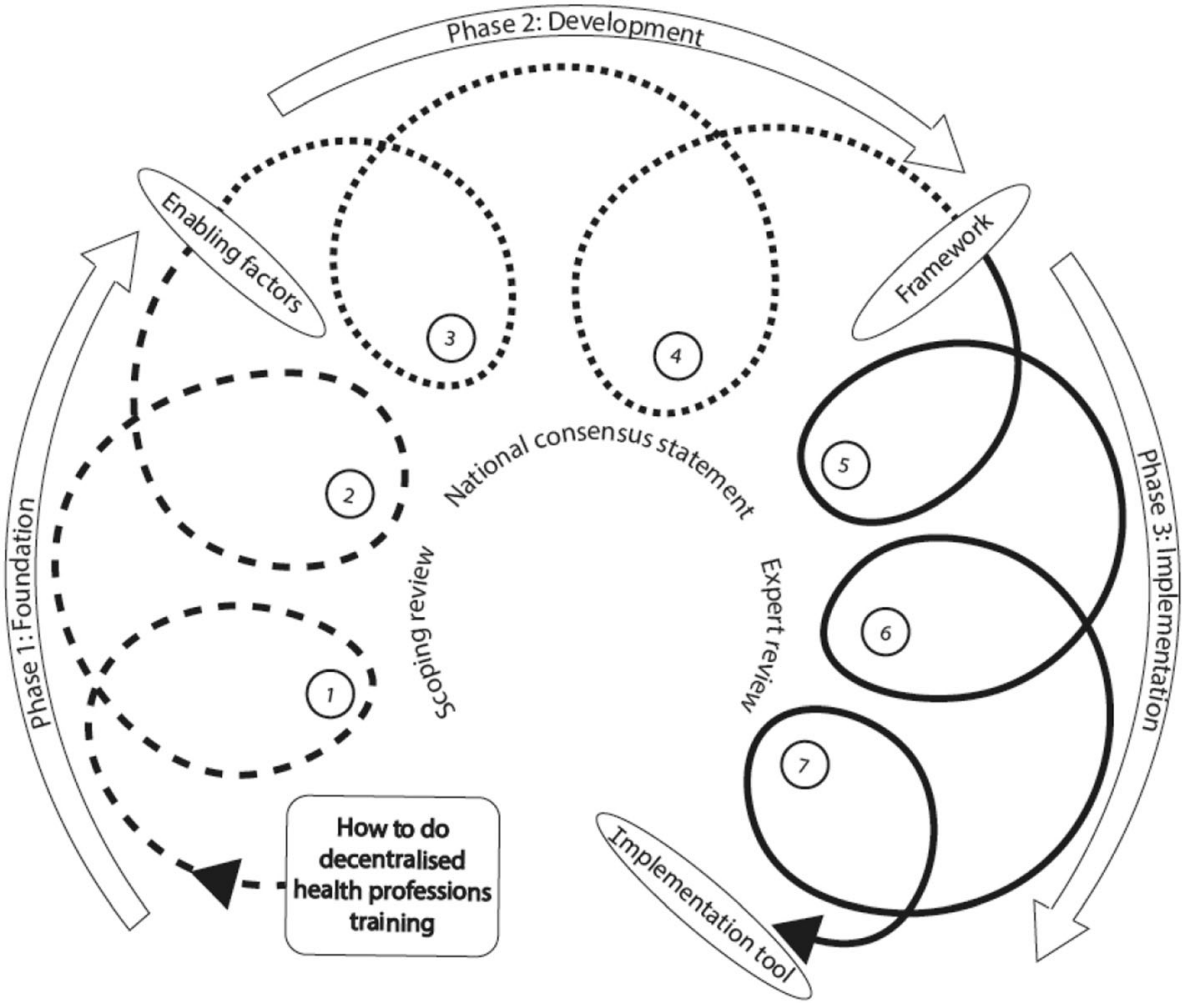
A visual representation of the evolution of the project. The numbered loops represent the 7 PAR workshops. The outer arrows highlight the three phases (also identified by the dotted and continuous lines) that framed the development of the project. The inner text reflects key activities that informed each phase. The text placed within the oval shapes points to key outputs of each project phase

In summary, the process across the three phases has been characterised as iterative and complex. Each new engagement with stakeholders often took us several steps back before it took us forward. Fig. 2 offers a visual representation of the project’s evolution showing the iterative cycles – the feedback loops described earlier - through the PAR episodes that enabled us to progress from the initial question of how to do distributed health professions training, to the development of the Framework and a Tool to facilitate its implementation (Additional file 2).

## Discussion – a framework to influence practice and inform policy

Our intention with this project was to develop a framework that could inform and guide the establishment and implementation of health professions training at distributed clinical sites in SA. In reflecting on the process that we followed, we drew on literature that explores how knowledge comes into being, including in policy contexts. Our scoping review had enabled us to first pay attention to the existing scholarship in the field, applying what Laksov and colleagues [31] framed as a ‘distanced perspective’, perusing available evidence to detect similarities and differences that could direct future work. However, recognising that patterns would not seamlessly emerge from the literature to fit into a framework [32], we made an active choice as researchers [31] to involve stakeholders as participants in a PAR process so that the interpretation of the literature and application to real-life contexts would be derived from the lived experience [26] of a community of inquiry. Thus we drew on the thinking and practices of key stakeholders in the field – the external voices - through our extensive consultative process.

Using such an approach was important in terms of the complexity of the system in which we sought to examine DHPT without being reductionist, focusing on interactions between components in the Framework rather than on the individual components themselves. This arises from complexity theory, where the inherently complex properties of a system emerge from the relationships that exist amongst its components [21] (thus affirming the centrality of relationships in our framework), and are open to external influences that can lead to system change, and thus changes in policy and practice.

The approach of applying externally produced knowledge about a particular topic as a lens through which the prevailing literature can be considered and ultimately taken up in policy, has been previously described [33]. In their work on knowledge in policy, Freeman and Sturdy [33] developed a schema that suggested three knowledge forms: embodied, inscribed and enacted. All of the participants brought with them their ‘embodied’ knowledge relating to aspects of distributed training. Also sometimes called ‘embrained’ knowledge, embodied knowledge has both a practical, tacit element, often manifesting in skills (know how), as well as a verbal element (know that). Thus participants’ embodied knowledge represented their individual, lived experiences as framed in their day-to-day lives, as well as their understanding or experience of existing models.

Knowledge can, however, also be inscribed either in writing or through the generation of other artefacts such as drawings, diagrams and the like. The scoping review highlighted the wealth of existing inscribed knowledge relating to DHPT, available internationally and locally. In this project, there were multiple incidents of further inscription such as the group activities during the workshops, where participants were encouraged to generate visual representations of the ideal distributed learning context; the development of the workshop reports and subsequent publication of these reports; the expert review process and, ultimately, the development of the national consensus statement. Inscribed knowledge is important as it ‘provides a corrective to the instability and fragility of human bodies and memories’ [33] and it was a key product of our work.

While the generation of both the embodied and the inscribed knowledge was fundamental to the development of the Framework, it could be argued that the extent to which this work becomes enacted knowledge, that ‘knowledge generators’ become ‘knowledge users’ [25] could serve as a litmus test for its value. Hence the development of the Implementation Tool and its application in different settings. We recognise that it is only when the knowledge is enacted that the embodied and inscribed knowledge achieves standing and significance, in this case in relation to policy and practice. Importantly, the enacted knowledge is not static for as the knowledge, in our case the Framework, becomes enacted through using the Tool, gaps will be identified, interpretations will become concretised, and new ideas will be generated. In the context of this work, therefore, these artefacts are offered as guidelines for enaction, with the caveat that such enaction, given the complexity inherent within the framework, will be influenced by a ‘degree of interpretive flexibility’ [33] that will require ongoing interaction across the knowledge community from which it has emerged. In essence, this means that all who have contributed to this project share responsibility to ensure its application and renewal. With this article we hope to extend the dissemination of the Framework and the Tool.

There are also limitations to this study. There was no involvement of service users (grassroot communities) in the development of the Framework, mainly due to our focus on the community of health professions educators, service providers and policy makers. Furthermore, while there was some student involvement in the piloting of the Implementation Tool, there was limited student participation in the workshops. However, many of the participants had extensive engagement with students in their contexts, including members of the research team [34].

Use of the Tool in various settings is expected to take community perspectives into account, in line with key principles underlying the Framework such as social accountability and shared vision. We further recognise that those who contributed to the development of the framework would probably have been motivated by a desire to see the successful implementation of DPHT and therefore may have been less critical than those who are not supportive of this approach. We acknowledge that the Tool is in many ways aspirational and that its implementation could encounter challenges when faced with local contexts; nevertheless, it provides a benchmark that can inform planning. Finally, despite the initial project’s country-specific focus, the applicability of the Framework and Tool for effective DHPT in other national contexts has subsequently been explored at international meetings, generating positive feedback and interest. This points to an opportunity for future, multi-country research.

## Conclusion

It could be argued that PAR is premised on the notion that evidence can be generated from within and that through the interactions between the different role-players, new understandings emerge [27]. Next steps include further synthesis with the existing body of knowledge that can serve to strengthen such evidence. Work of this nature, however, takes time and is complex. Nevertheless, the outcome of this process, the Framework and its subsequent implementation, has enabled us to not only respond to calls for application of evidence in our educational practice, but also to begin to influence policy and enhance practice across many levels of stakeholders at a national level. Additional collaborative initiatives that will build on these early successes are needed going forward.

## Supplementary information

**Additional file 1.**

**Additional file 2.**

## Data Availability

The datasets and materials are available from the corresponding author on request,

## Abbreviations

CDC: Centers for Disease Control and Prevention
DPHT: Distributed health professions training
HPCSA: Health Professions Council of South Africa
HPE: Health professions education
NDoH: National Department of Health
PAR: Participatory action research
SA: South Africa
SAAHE: Southern African Association of health educationalists
SUCCEED: Stellenbosch University Collaborative Capacity Enhancement through Engagement with Districts
SU FMHS: Stellenbosch University Faculty of Medicine and Health Sciences
UKZN: University of KwaZulu-Natal
WSU FHS: Walter Sisulu University Faculty of Health Sciences
