# Supplementary material for: A framework for distributed health professions training: using participatory action research to build consensus
Source: BMC Med Educ. 2020 May 14;20:154. doi: 10.1186/s12909-020-02046-z (PMC7227246; doi:10.1186/s12909-020-02046-z)
Supplement: Supplementary file 1 — Additional file 1. [file 12909_2020_2046_MOESM1_ESM.docx]

**Table 2: Essential components and their enabling factors (unabridged version)**

| Leadership and governance influences effective DHPT, through the decision-making processes and roles and responsibilities of stakeholders. |
| --- |
| 1. All stakeholders, including the health services, community, and training institutions, engage in mutually beneficial and equitable partnerships. 2. The roles and responsibilities of training institutions, health services, and communities, are clear to everyone involved. 3. All levels of management are committed to effective collaboration to support students’ learning. 4. Senior management of all stakeholders demonstrate collaborative and visionary leadership toward a shared purpose. 5. Champions take responsibility for distributed training. 6. Funding for training initiatives is made available through a transparent funding model. 7. Formal and informal communication channels exist across all levels and among multiple stakeholders. 8. Monitoring, evaluation, and research on distributed training initiatives are encouraged by leadership. 9. The training institution:    - implements institutional policies that support distributed training.    - supports and capacitates primary supervisors and other site staff involved with students (see also factor 31).    - builds and maintains relationships with the site.    - selects students most likely to practice in rural and remote areas.    - becomes familiar with each site’s strengths and challenges. |
| The curriculum provides the scaffolding that informs the learning outcomes, content, mode of delivery, and assessment of students, and evaluation of the curriculum itself. |
| 10 Management of the training institution takes leadership in prioritising and implementing distributed training programmes.  11 Consistency amongst learning outcomes across training institutions is required for students to learn together at the same site.  12 Learning outcomes for distributed training include a focus on:   - Social determinants of health. - Common, undifferentiated problems in primary health care. - An integrated spectrum of health and illness. - Cultural awareness.   13 The curriculum for distributed training uses:   - Various teaching and learning approaches (e.g., student-centered, interprofessional, competency-based, self-directed, debriefing and reflection). - A patient-centered approach to care. - Opportunities for developing a range of competencies. - Flexibility to adapt to the realities of the individual site. - On-site, integrated and continuous student assessment.  1. Distributed training rotations should be of sufficient length to allow for immersion and integration for students, and continuity for the site.   15 Provision is made for regular and structured feedback from and to students.  16 Applicability of learning outcomes is assured by continuous monitoring, review, and modification of the curriculum. |
| The community is defined as the population that utilises the local health facility where students are trained, and is the reference point for the curriculum. |
| 1. Community stakeholders are identified and engaged. 2. Strong partnerships are forged and maintained with community stakeholders at the training site. 3. The community is involved in and supports the shared vision for the training initiative that meets their needs. 4. Students and staff are aware of and oriented to community needs. 5. Learning opportunities are available wherever health services are provided in the community, including home-based care. 6. Students learn through being immersed in the community. 7. Stakeholders engage in collective celebration of accomplishments. |
| The training environment includes (a) people who work at the distributed training site, and in the community, contributing to the training of the students; and (b) the training site as the context and physical environment within which the distributed training takes place. |
| (a) people   1. There is a dedicated person at the training site who coordinates the training and communicates with the training institution. 2. Staff from various professions work with students at the site to facilitate their learning. They are provided opportunities to learn how to teach, developing an understanding of the importance of role modelling, resilience and professionalism. 3. Before students arrive, staff at the site receive the information they need about learning outcomes and relevant guidelines to support students’ learning. 4. Site staff who train students receive recognition from the training institution. 5. Site staff provide feedback about student performance. 6. Subject specialists support distributed training through regular outreach visits. 7. At least one health professional is motivated and available to act as primary supervisor for students. 8. The primary supervisor:  - develops, implements, and evaluates the training at the site. - is involved in formative and summative assessment of students. - receives the necessary support and training technologies. - develops her/his own capacity in teaching and learning, which is made available by the training institution.   (b) Place   1. The training site is selected collaboratively by stakeholders, including service providers, training institution, site management, and relevant others.   33 Site selection is based on patient profile, learning outcomes, quality of care, and other factors that will provide relevant learning opportunities.  34 Medical equipment, appropriate to the level of care of the facility and to the required learning outcomes, is available.  35 Sufficient space for training activities is made available.  36 Materials to enhance learning are made available on-site, preferably through internet connectivity and information technology equipment.   1. Depending on location of the site, accommodation and transport for students are made available. Include community outreach and the use of community resources where appropriate. |
| The students are learners enrolled for any programme in health professions at a training institution. |
| 1. Students:    - receive orientation before they begin a rotation.    - have academic and social support available when and where they need it.    - provide feedback after they complete a rotation.    - have adequate arrangements for safety and security.   39 Student-staff ratios are mutually agreed upon during site selection.  40 At least two students are assigned to a site to ensure peer engagement.  41 Reasonable logistical arrangements are made by the training institution. |
