# Supplementary material for: A framework for distributed health professions training: using participatory action research to build consensus
Source: BMC Med Educ. 2020 May 14;20:154. doi: 10.1186/s12909-020-02046-z (PMC7227246; doi:10.1186/s12909-020-02046-z)
Supplement: Supplementary file 2 — Additional file 2. [file 12909_2020_2046_MOESM2_ESM.docx]

**IMPLEMENTATION TOOL**

**Applying the Framework for Effective Distributed Health Professions Training**

**Simple Rules Rubric**


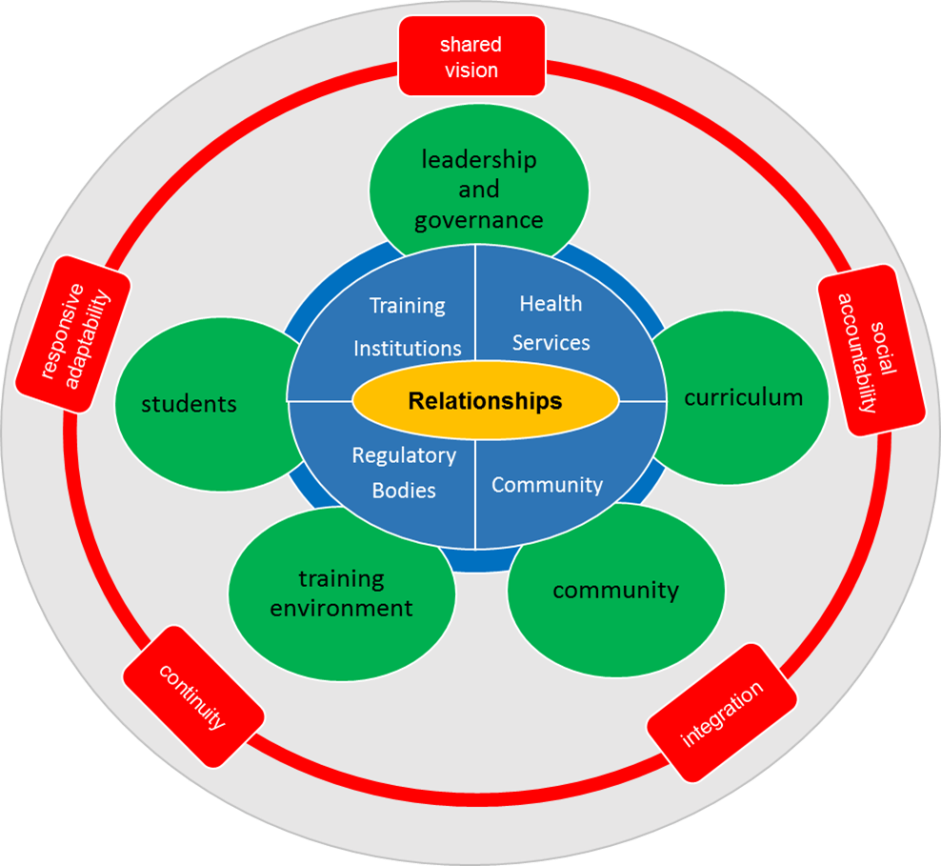
Every system will have a unique implementation of the Framework for Effective Distributed Health Professions Training. The most effective implementation for your institution will depend on the maturity of your program, the number of institutions involved, the geographical distribution, the number of students and disciplines represented, and many other factors. While many variations are to be expected, evidence shows that effective programmes share patterns of performance, which are represented in the enabling factors of the Framework for Effective Distributed Health Professions Training.

Complexity of the training context and the number of stakeholders involved make it difficult to plan for, apply, and evaluate all the individual enabling factors at the same time. A short list of simple rules can be used to organise the factors, simplifying the process whilst continuing to set conditions for patterns of effective teaching and learning. These Simple Rules are:

1. Build and maintain relationships
2. Move toward a shared vision
3. Fulfill roles and responsibilities
4. Balance needs and provide support
5. Engage with learning
6. Evaluate and provide feedback

The Simple Rules Rubric organises the enabling factors to help you plan for, implement, and evaluate changes in your program over time.

*Your Turn: Consider your institution and its current distributed training program. WHAT? What is the current status for each of the enabling factors? SO WHAT? So what are reasonable options for action to improve? NOW WHAT? What activities are good next steps to move your program forward?*

| 1. Build and maintain relationships   Many role players are involved in distributed training in the health professions, and all of them are important to the success of the programme. It is good to invest in relationships early and continuously. | **Not at all** | **Somewhat** | **To a great extent** | **Not applicable** | **Next Steps** |
| --- | --- | --- | --- | --- | --- |
| 1. All stakeholders, including the health services, community, and training institutions, engage in mutually beneficial and equitable partnerships. |  |  |  |  |  |
| 1. Formal and informal communication channels exist across all levels and among multiple stakeholders. |  |  |  |  |  |
| 1. The training institution builds and maintains relationships with the site. |  |  |  |  |  |
| 1. Community stakeholders are identified and engaged through strong partnerships. |  |  |  |  |  |
| 1. There is a dedicated person at the training site who coordinates the training and communicates with the training institution. |  |  |  |  |  |
| 1. Site staff who train students receive recognition from the training institution |  |  |  |  |  |

###

| 1. Move toward a shared vision   All stakeholders should understand and commit to working toward a shared vision for distributed training in the health professions across multiple perspectives. | **Not at all** | **Somewhat** | **To a great extent** | **Not applicable** | **Next Steps** |
| --- | --- | --- | --- | --- | --- |
| 1. Management is committed to effective collaboration to support students’ learning. |  |  |  |  |  |
| Senior management of all stakeholders demonstrate collaborative and visionary leadership toward a shared purpose. |  |  |  |  |  |
| 1. At all different levels in all institutions, champions are appointed and take responsibility for distributed training. |  |  |  |  |  |
| 1. The community is involved in and supports the shared vision for the training initiative that meets their needs. |  |  |  |  |  |
| 1. Students and staff demonstrate a community orientation and awareness. |  |  |  |  |  |
| 1. The training institution implements institutional policies that prioritise distributed training. |  |  |  |  |  |

| 1. Fulfill roles and responsibilities   Each role player has specific responsibilities and effective distributed training for the health professions requires them to fulfill their commitments. | **Not at all** | **Somewhat** | **To a great extent** | **Not applicable** | **Next Steps** |
| --- | --- | --- | --- | --- | --- |
| 1. The roles and responsibilities are clear for the training institution, health service, and community. |  |  |  |  |  |
| Training institution selects students most likely to practice in distributed areas (relevant for training institution leaders). |  |  |  |  |  |
| Management of the training institution takes leadership in prioritising and implementing programmes. |  |  |  |  |  |
| Different training institutions using the same training site have consistent learning outcomes (relevant for training institution management). |  |  |  |  |  |
| Subject specialists support distributed training through regular outreach visits. |  |  |  |  |  |
| At least one health professional is motivated and available to act as primary supervisor for students. |  |  |  |  |  |
| The primary supervisor:   - develops, implements, and evaluates the training at the site. - develops her/his own capacity in teaching and learning, which is made available by the training institution. |  |  |  |  |  |

| 1. Balance needs and provide support   All stakeholders make contributions and receive support throughout the distributed training process. | **Not at all** | **Somewhat** | **To a great extent** | **Not applicable** | **Next Steps** |
| --- | --- | --- | --- | --- | --- |
| 1. Funding for training initiatives is made available |  |  |  |  |  |
| The training institution:   - supports and capacitates primary supervisors and other site staff involved with students. - becomes familiar with each site’s strengths and challenges. |  |  |  |  |  |
| Staff from various professions work with students at the site to facilitate their learning, and are provided opportunities to learn how to teach |  |  |  |  |  |
| The training site is selected collaboratively by stakeholders, including service providers, training institution, site management, and relevant others. |  |  |  |  |  |
| Depending on location of the site, accommodation and transport for students are made available, including the use of community resources where appropriate. |  |  |  |  |  |
| Students:   - receive orientation before they begin a rotation. - have academic and social support available when and where they need it. - have adequate arrangements for safety and security. |  |  |  |  |  |
| Student-staff ratios are agreed upon by all stakeholders |  |  |  |  |  |

| 1. Engage with learning   Students' learning should be the primary focus for all facets of the distributed training programme. | **Not at all** | **Somewhat** | **To a great extent** | **Not applicable** | **Next Steps** |
| --- | --- | --- | --- | --- | --- |
| 1. Learning outcomes for disitributed training include a focus on:  - Social determinants of health. - Common, undifferentiated problems in primary health care. - An integrated spectrum of health and illness. - Cultural awareness. |  |  |  |  |  |
| The curriculum for distributed training uses:   - Various teaching and learning approaches (e.g., student-centered, interprofessional, competency-based, debriefing and reflection). - A patient-centered approach to care. - Opportunities for developing a range of competencies. - Flexibility to adapt to the realities of the individual site |  |  |  |  |  |
| Distributed training rotations should be of sufficient length to allow for community immersion and integration for students and continuity for the site |  |  |  |  |  |
| Learning opportunities are available at all levels in the community, including home-based care. |  |  |  |  |  |
| Before students arrive, staff at the site receive the information they need about learning outcomes and relevant guidelines to support students’ learning. |  |  |  |  |  |
| Site selection is based on patient profile, learning outcomes, quality of care, and other factors that will provide relevant learning opportunities |  |  |  |  |  |
| Medical equipment, appropriate to the level of care of the facility and to the required learning outcomes, is available. |  |  |  |  |  |
| Sufficient space for training activities is made available. |  |  |  |  |  |
| Materials to enhance learning are made available on-site, preferably through internet connectivity and information technology equipment. |  |  |  |  |  |
| At least two students are assigned to a site to ensure peer engagement. |  |  |  |  |  |

| 1. Evaluate and provide feedback   Feedback from and evaluation of stakeholders ensures programme quality and continuous improvement. | **Not at all** | **Somewhat** | **To a great extent** | **Not applicable** | **Next Steps** |
| --- | --- | --- | --- | --- | --- |
| 1. Monitoring, evaluation, and research on training initiatives are supported by leadership. |  |  |  |  |  |
| The curriculum uses integrated and continuous student assessment. |  |  |  |  |  |
| Provision is made for regular and structured feedback from and to students, including after completion of a rotation. |  |  |  |  |  |
| Applicability of learning outcomes is assured by continuous monitoring, review, and modification of the curriculum. |  |  |  |  |  |
| Stakeholders engage in collective celebration of accomplishments. |  |  |  |  |  |
| Site staff provide feedback about student performance. |  |  |  |  |  |
| The primary supervisor is involved in formative and summative assessment of students. |  |  |  |  |  |
